# Chagas disease as an underrecognized cause of stroke: implications for public health

**DOI:** 10.3389/fmed.2024.1473425

**Published:** 2024-11-22

**Authors:** Jorge Vásconez-González, Camila Miño, Camila Salazar-Santoliva, Melissa Villavicencio-Gomezjurado, Esteban Ortiz-Prado

**Affiliations:** ^1^One Health Research Group, Faculty of Health Science, Universidad de Las Américas, Quito, Ecuador; ^2^Department of Public Health, London School of Hygiene and Tropical Medicine, London, United Kingdom

**Keywords:** Chagas disease, stroke, public health, *Trypanosoma cruzi*, cerebrovascular accident

## Introduction

Chagas disease (CD) is endemic in 21 countries in Latin America, affecting around 30,000 people annually and putting approximately 70 million individuals at risk (1, 2). Factors such as poverty, inadequate housing, and insufficient health infrastructure contribute to its high prevalence within the region (1, 3). Stroke, on the other hand, impacts more than 15 million people globally each year, causing 5 million deaths and leaving another 5 million permanently disabled (4). This number is expected to increase by 2025. By 2050, 77.6% of disability-adjusted life years (DALYs) will be caused by non-communicable diseases (5). The relationship between Chagas disease and stroke has been poorly studied, but both conditions pose significant public health challenges due to their high morbidity and mortality rates in Latin America. For instance, in this region alone, Chagas disease accounts for 662,000 disability-adjusted life years (DALYs), while stroke contributes to 20 million DALYs (6–8).

The complex relationship between these two pathologies must be analyzed together because, although complications of Chagas disease such as megacolon, megaesophagus, and cardiomegaly are well-documented (9–13), the link between CD and stroke has not received adequate attention. Oliveira-Filho outlines mechanisms through which CD can lead to cardioembolic stroke, including chronic inflammation, activation of the coagulation system, cardioembolism, microembolism, and watershed infarcts from low cardiac output states (14). Additionally, patients with CD can experience non-embolic strokes due to small vessel disease, atherosclerosis, and cryptogenic causes (15).

**Figure 1:**
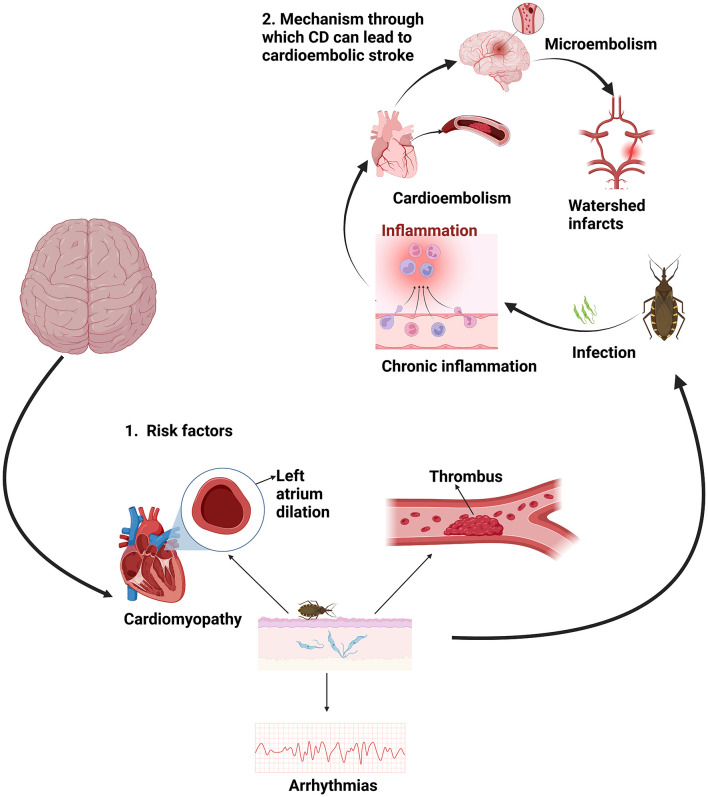
Main risk factors and mechanism of stroke in CD. Created with BioRender.com.

The mechanisms by which a parasitic infection like Chagas disease increases the risk of developing a stroke are not entirely clear. However, several proposed mechanisms exist (Figure 1). Carod-Artal et al. found that approximately 40% of Chagas disease (CD) patients with ischemic stroke have an apical aneurysm of the left ventricle (16). Additionally, 70% of these patients exhibit electrocardiogram abnormalities, with right bundle branch block present in 35% of cases, left anterior fascicular block in 17%, and atrial fibrillation in 15% (16). This may be one of the primary reasons why Chagas disease can increase the risk of developing a stroke. Furthermore, family history is also significant, as 32.7% of CD patients who suffer a stroke have relatives with Chagas disease. Special caution is necessary in managing these patients to prevent stroke (17).

In some areas where Chagas disease (CD) is highly prevalent, stroke could be the first manifestation of CD, particularly in patients with left ventricular dysfunction. In regions like Brazil, 40% of patients diagnosed with CD have been identified after suffering a stroke (18). About 38.4% of patients with Chagas-related stroke have asymptomatic T. cruzi infection, which can lead to small and large vessel infarctions (19). Asymptomatic patients may also present with apical aneurysm, left atrial dilation, and left ventricular hypokinesia, increasing the risk of cardioembolism. With the aging population, cerebrovascular complications in CD patients are expected to rise (16).

Cerqueira-Silva et al. conducted a study involving 210 patients with heart failure and CD and 194 patients with heart failure without CD. They found that the incidence of stroke was 20.2 per 1,000 patient-years in the CD group and 13.9 per 1,000 patient-years in those without CD. Additionally, it was revealed that Chagas disease is an independent predictor of first ischemic stroke (HR 2.54, 95% CI: 1.01–6.42) (20). In a study conducted in Brazil comparing 329 patients with Chagas cardiomyopathy to 461 patients with non-Chagas cardiomyopathy, the mean age was 58.3 years. It was observed that strokes were more frequent in the group with CD compared to the infection-free group (17.3% versus 11.1%; p < 0.01). Additionally, Chagas patients who experienced a stroke had a very high frequency of individuals without any vascular risk factors (40.4%; OR, 4.8) (21).

In Colombia, a case-control study involving 82 cases (mean age 66.6) and 102 controls (mean age 61.9) revealed that T. cruzi infection was more frequent and statistically significant in stroke cases (24.4%) compared to controls (1.9%) (*p* < 0.00001). Even after excluding Chagas patients with cardiac abnormalities, the significance remained (*p* < 0.05) (22). In a case-control study in Brazil, T. cruzi infection was significantly more common in 101 consecutive cases of acute stroke than in 100 cases of acute coronary syndrome (14% vs. 2%; *p* = 0.002) (23).

Whether stroke is diagnosed first and then CD identified, or vice versa, it is crucial to note that having Chagas disease significantly increases the risk in stroke patients (24). Lima-Coste et al. followed 1,398 patients over ten years and found that the risk of death from stroke in CD patients was twice that of uninfected individuals (adjusted hazard ratio, 2.36; 95% CI, 1.25 to 4.44) (24). In this study, elevated levels of type B natriuretic peptide in these patients were determined to be predictors of mortality, especially when combined with atrial fibrillation, which increased the risk of death by 11.49% (24). It is estimated that more than 60% of stroke patients with Chagas disease living in endemic regions have a family history of the infection, compared to only 16% of stroke patients without Chagas disease. In addition to having relatives with Chagas disease and having lived in an adobe house during childhood, these social variables are associated with Chagas-related strokes (*p* < 0.001) (25).

Additionally, it has been reported that both chronic heart disease and gastrointestinal forms of CD can coexist in patients who have experienced a Chagas-related stroke. Chronic intestinal CD has been identified in 20% of these patients, with 8% having megaesophagus, 8% with megacolon, and 4% with both conditions (25). Regarding heart disease, left ventricular dysfunction has been observed in 65% of stroke patients, apical aneurysm in 40%, and left ventricular dilation in 23% (25).

## Call for action

The information about this not-so-rare link is vital for understanding that a parasitic disease like Chagas disease can leave severe sequelae that significantly increase the risk of stroke. Therefore, it is crucial to improve screening campaigns and identify risk factors. Given the above, diagnosing CD in stroke patients, particularly those from endemic areas or with relevant travel histories, is essential.

Educational programs for both the public and healthcare providers are crucial, as awareness of the stroke risk in Chagas patients is only 5% (18).

Moreover, diagnostic campaigns are needed, as less than 10% of infected individuals are aware of their condition. Early detection of CD can lead to a 100% cure rate, while untreated cases may result in severe, often irreversible complications affecting various systems (26).

Raising awareness, enhancing detection efforts, and providing comprehensive education can prevent many stroke cases related to Chagas disease and improve overall patient outcomes. As shown in Table 1.

**Table 1:** Recommendations for the management of chagasic stroke.

**Research**	**Clinical settings**	**Public Health**
- Development and testing of better treatment regimens, including new medications (27). -Development of less toxic drugs (27). -Development of better methods to evaluate the effectiveness of treatment (27). -Create multicenter database on chagas patients (16). - Carry out epidemiological studies on CD due to T cruzi strains for correct taxonomic identification, in addition to studies to determine their geographical areas (16, 28).	-Standard diagnostic procedures (29). -Effective treatment strategy (29). -Detection of *T cruzi* in patients with stroke in endemic regions (16). -Standardization of follow-up of patients with chronic CD (16). -Active surveillance programs among asymptomatic patients infected by *T cruzi* (16). -Development of Chagasic stroke prevention programs (16). -Detection of drug resistance of T. cruzi (16).	-Development of integrated and sustainable vector control protocols (27). -Improve monitoring and estimation of the real burden of disease (29). -Increase spray coverage and monitor insecticide resistance (29). -Develop active screening programs for *T cruzi* in stroke patients from endemic areas (16). -Coordination between national, regional and international policy makers, public health professionals and academics (29). -Assist community members in housing reconstruction projects in rural areas to prevent Chagas disease by reducing exposure to vectors (28).

Further research should focus on developing better treatment regimens and creating a multicenter database on Chagas patients (16, 22). Clinical settings need standard diagnostic procedures and effective treatment strategies, particularly in endemic regions (16, 24). Public health initiatives must include integrated vector control protocols and active screening programs for T. cruzi (16, 22, 24). Coordination between national and international stakeholders and community involvement in preventive measures are also essential (23, 24). By implementing these recommendations, we can significantly reduce the burden of Chagas-related strokes and enhance patient care.
